# Social wellbeing, loneliness, and symptom burden in head and neck cancer survivors: a latent class analysis

**DOI:** 10.1080/07347332.2025.2565302

**Published:** 2025-10-03

**Authors:** Eden R. Brauer, Kristen R. Choi, Laura Petersen, Patricia A. Ganz, Maie A. St. John, Deborah J. Wong, Emily J. Martin

**Affiliations:** a School of Nursing, University of California, Los Angeles (UCLA), Los Angeles, California, USA;; b Multidisciplinary Head and Neck Cancer Program, University of California, Los Angeles (UCLA), Los Angeles, California, USA;; c Department of Health Policy and Management, Fielding School of Public Health, University of California, Los Angeles (UCLA), Los Angeles, California, USA;; d Division of Hematology/Oncology, Department of Medicine, David Geffen School of Medicine at UCLA, University of California, Los Angeles (UCLA), Los Angeles, California, USA;; e Department of Head & Neck Surgery, David Geffen School of Medicine at UCLA, University of California, Los Angeles (UCLA), Los Angeles, California, USA;; f Palliative Care Program, Department of Medicine, David Geffen School of Medicine at UCLA, University of California, Los Angeles (UCLA), Los Angeles, California, USA

**Keywords:** Cancer survivorship, head and neck cancer, loneliness, social wellbeing, symptom clusters

## Abstract

**Purpose:**

To identify patterns of co-occurring symptoms in a sample of head and neck cancer (HNC) survivors; compare symptom burden among latent classes; and examine associations between symptom classes and social wellbeing outcomes.

**Methods:**

This cross-sectional survey of HNC survivors ≥1 year post-diagnosis was conducted in 2020 using a tumor registry at an academic medical center. Primary outcomes were loneliness and activities impairment. Participants reported 19 HNC-specific symptoms using the European Organization for Research and Treatment of Cancer HNC module (EORTC hN-43), and general cancer symptoms (sleep, pain, anxiety, depression, fatigue). Latent class analysis was used to identify subgroups with different symptom patterns. Multivariable regression models were estimated to examine associations between class membership and social wellbeing outcomes.

**Findings:**

Three hundred forty-seven survivors (mean age 65.5 ± 11.3 years; 3.72 ± 2.3 years post-diagnosis) completed the survey. Participants were predominantly male (72.6%), White (81.6%), and under age 65 years at diagnosis (59.4%). Three symptom classes were identified: (1) complex symptom burden (45%), (2) oral/sensory symptom dominant (38.9%), and (3) limited symptom impact (15.8%). Membership in the complex symptom burden class was associated with increased activity impairment (*β* = 28.6, *SE* = 3.7, *p*<.001) and increased loneliness (*β* = 1.1, *SE* = 0.2, *p*<.001) compared to the oral/sensory class (most similar to overall sample).

**Conclusion:**

Elevated and complex HNC symptom burden is associated with higher levels of general cancer symptoms and risk for loneliness and reduced engagement in daily activities. Tailored survivorship care models addressing symptom profiles of HNC survivors, particularly those with complex symptom burden, are needed to improve quality of life.

## Introduction

Loneliness is defined as a subjective, distressing experience arising from perceived social isolation or unmet needs related to interpersonal connection.^1,2^ Conceptualized as a biopsychosocial stressor, loneliness is associated with profound physiological and psychological impacts, including elevated levels of systemic inflammation that can compromise cardiovascular, immune, and neuroendocrine processes.^3–6^ Notably, loneliness is increasingly recognized as a public health concern, with the United States (US) Surgeon General reporting that loneliness and social disconnection can increase the risk for premature death by 26%, and have detrimental effects to health that are comparable to smoking 15 cigarettes daily.^4,7^

For individuals living with or beyond cancer, the experience of both the diagnosis and its treatments can result in long-term physical and emotional effects that can contribute to loneliness and social disconnection.^8–12^ Cancer survivors often report disruptions in social roles and identities, challenges in navigating existing and new social relationships, and feelings of isolation that frequently extend beyond initial diagnostic and treatment phases.^12,13^ Higher levels of loneliness in cancer survivors have been associated with a range of adverse health outcomes, including psychological distress, cognitive impairment, chronic pain, and increased mortality risk.^8,13–15^ With the number of cancer survivors in the U.S. projected to reach 26 million by 2040,^16^ better understanding of the dynamics between chronic symptoms, treatment effects and dimensions of social wellbeing is warranted.

When compared to the general population and other cancer populations, individuals with head and neck cancers (HNC) experience higher rates of loneliness, in part because of the anatomical structures affected and their associated functions, such as communication, eating, and facial expressions.^17–22^ HNC survivors face unique challenges, including disfigurement, scarring, pain, xerostomia, dysphagia, dysgeusia, nerve damage, and loss of teeth. These, in turn, can result in complex speech and swallowing difficulties and vulnerability to cancer-associated impacts on daily activities and social wellbeing.^20,23–26^ Social stigma and concerns about self-image can exacerbate these challenges. Prior research has highlighted the extreme isolation HNC survivors experience due to avoidance of social activities, such as eating alone to spare others from witnessing them regurgitate or waiting until dark to take a walk outside.^27^ In fact, the loneliness of living with HNC has been described as “a profile in captivity.”^28^ With reduced participation in celebrations, family meals, and outings, HNC patients report diminished enjoyment in social eating and increased difficulty engaging in conversations in social environments.^26^ Even at home, HNC patients report a loss of togetherness, particularly during mealtime, and often dine separately due to the time-consuming process of eating and the discomfort they cause their loved ones.^29,30^ Despite these alarming accounts, few studies have explored specific patterns of cancer-related symptoms and treatment effects and how they relate to social outcomes among HNC survivors.

To address these gaps, the purpose of this study was to (1) identify patterns of co-occurring symptoms among HNC survivors using latent class analysis, (2) compare symptom burden among latent classes, and (3) examine associations of class membership to two dimensions of social wellbeing: loneliness and reduced activity levels. By exploring these associations, this research seeks to provide a more comprehensive understanding of the interconnections between symptom burden and social wellbeing, ultimately informing more targeted and effective interventions to improve quality of life for HNC survivors.

## Patients and methods

### Design and sample

This was a cross-sectional survey conducted in 2019 to early 2020. After obtaining approval from the Institutional Review Board, we identified and recruited individuals from the medical center’s tumor registry. To be eligible, individuals were 18 years or older and more than one year post-diagnosis of HNC. HNC diagnoses included cancers of the nasopharynx, oropharynx, hypopharynx, larynx, tonsil, salivary glands, tongue, lip and oral cavity. Individuals who were unable to complete the survey in English were excluded. In total, 1173 potentially eligible participants were identified and invited to participate in the study *via* email (when available) or postal mail, with a maximum of two follow-up attempts.

### Survey procedures

The survey was administered *via* Research Electronic Data Capture (REDCap), a web-based platform, with hardcopy options when preferred.^31,32^ The comprehensive survey took approximately 60 to 90 min to complete and included multiple validated questionnaires. The questionnaires used in this analysis are described below.

### Measures

#### Outcomes

Outcome variables were loneliness and activity impairment. Loneliness was assessed using the validated 3-item UCLA Loneliness Scale, with a possible score range of 3–9, in which higher scores indicate more loneliness.^33,34^ The tool’s questions evaluate perceived social connection, feelings of isolation, and sense of disconnection from others. Activity impairment was assessed using the 6-item Work Productivity and Activity Impairment (WPAI): Specific Health Problem (SHP), version 2.0 questionnaire, which measures impairments in both paid work and unpaid daily activities and responsibilities.^35,36^ Given the high number of retirees in our sample, responses were used to calculate a WPAI-Activities score, interpreted as the percentage of impairment in regular activities due to a health problem, with higher scores indicating greater impairment. The WPAI has been validated to quantify activity impairments in numerous populations with chronic diseases, including cancer.^37–40^

#### Exposures

HNC-specific symptom burden was measured using the European Organization for Research and Treatment of Cancer (EORTC HN-43) module on HNC, specifically designed to target the needs, experiences, and quality of life of patients with HNC in the context of contemporary treatment regimens.^41,42^ The EORTC HN-43 measures 19 HNC-specific symptoms, with scores of 0–100 and higher scores indicating greater severity. We dichotomized each of the 19 EORTC HN-43 subscales into binary items based on presence or absence of the issue, where scores of 0 indicated absence and any score >0 indicated presence.

In addition to HNC-specific symptoms, the survey assessed general cancer-related symptom burden common across cancer types, including sleep disturbance, pain, anxiety, depression, and low energy or fatigue, referred to collectively as SPADE symptoms.^43,44^ Depression, anxiety, pain interference and fatigue were measured using Patient Reported Outcomes Measurement System (PROMIS) short forms, with raw scores converted to standardized *T*-scores (mean = 50, *SD* = 10) based on a reference population.^45–49^ Higher PROMIS scores indicate more of the construct measured. Subjective sleep quality and insomnia symptoms were assessed with the 7-item Insomnia Severity Index (ISI), with a possible range of 0–28 and higher scores indicating worse sleep.^50,51^ Sociodemographic and clinical data were collected to characterize the sample, including gender, race/ethnicity, marital status, insurance type, household income, educational attainment, age at HNC diagnosis, primary tumor site, and cancer treatment history.

### Data analysis

We used frequencies and descriptive statistics to characterize the sample based on demographic factors, clinical characteristics, and symptom burden. To identify latent symptom clusters, we applied latent class analysis (LCA) to the 19 EORTC items. LCA is a person-based approach to identifying unobserved heterogeneity in a population.^52^ In LCA, similar individuals are grouped together based on their responses to a set of observed input variables, generating a latent class variable for group membership. We enumerated latent classes using 19 dichotomous EORTC symptoms as input variables, starting with two classes and adding additional classes sequentially to determine the optimal number of classes. The models were compared to confirm the best-fit model that was both statistically supported and conceptually interpretable, using Bayesian Information Criterion (BIC), Akaike Information Criterion (AIC), Lo-Mendell- Rubin Likelihood Ratio Tests, and Chi-Square.^53^ Because BIC favors more parsimonious models, we prioritized BIC values.

After identifying the best-fit model based on number of latent classes, we assigned each participant to their most likely latent class based on class membership probabilities (i.e. latent class prevalence) and examined item response probabilities to characterize classes based on EORTC symptom profile. Item response probability is the probability of an indicator being present among members of a given latent class. Next, we used chisquare tests to compare differences in latent classes by SPADE symptom burden, as well as sociodemographic and clinical factors. Finally, we estimated two multiple linear regression models to examine the association of latent class membership to (1) activity impairment, and (2) loneliness scores. Models were adjusted for age of cancer diagnosis, gender, time since diagnosis, disease stage, comorbidities, and treatment history. F-tests were used to compare differences in each outcome variable across symptom clusters. Statistical differences were identified by *p* < .05. All analyses were conducted in R, version 2023.06.1 + 524.

## Findings

### Sample description

A total of 1,173 potentially eligible participants were identified from the tumor registry and invited to participate in the study. After invitation, an additional 26 individuals were found to be ineligible (23 deceased, 3 with no history of HNC), and 58 had incorrect contact information and were deemed unreachable. Of the remaining 1,089, 357 individuals responded to the survey invitation and 347 completed the survey, resulting in an overall response rate of 31.9% (347/1,089). Compared to respondents (*n* = 347), non-respondents (*n* = 799) were more likely to be non-White (*p*<.001), Hispanic (*p*=.002), and current smokers (*p*=.002). No significant differences were observed for age, gender, disease stage, time since diagnosis, marital status, or alcohol history.

**Figure 1.**
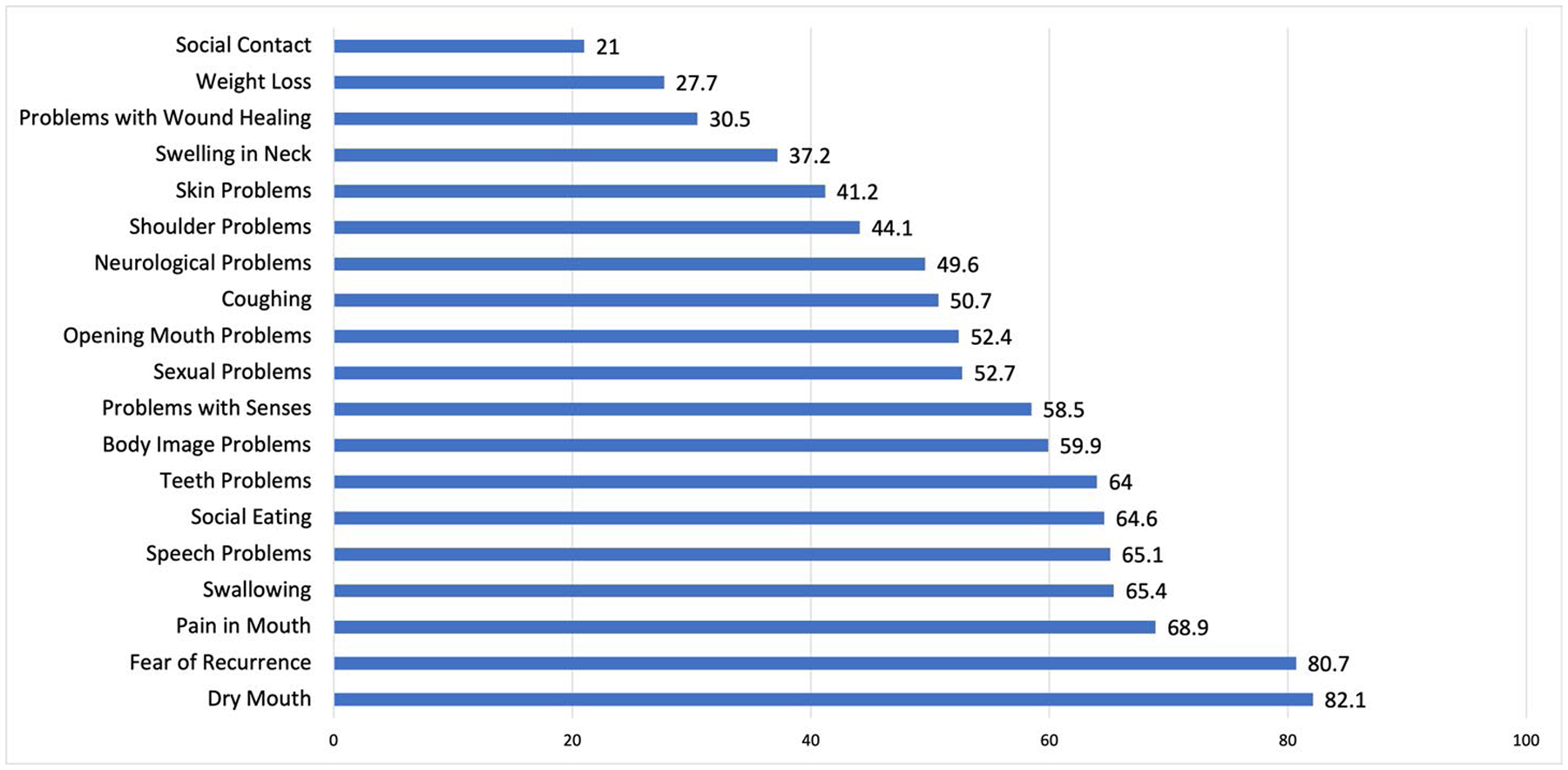
Percentage of participants reporting presence of HNC-specific symptoms. Presence of head and neck cancer (HNC) symptoms in a sample of 347 adults from Southern California, surveyed in 2020 from an academic medical center tumor registry. Symptom presence was measured using the European Organization for Research and Treatment of Cancer (EORTC HN-43) module on HNC, consisting of 19 subscales. Subscale scores were dichotomized into binary items based on presence or absence of the symptom.

Characteristics of the analytic sample of 347 respondents are shown in Table 1. Participants were primarily male (*n* = 252, 73%), identified their race/ethnicity as White (*n* = 283, 82%), and were married or partnered (*n* = 245, 71%). Medicare was the most frequent insurance type (*n* = 150, 43%). Most participants were diagnosed with HNC during middle adulthood at 40 to 64 years (*n* = 191, 55%) and had no comorbidities (*n* = 227, 65%). On average, participants reported 10 HNC-specific symptoms (*SD* = 5), with dry mouth (*n* = 285, 82.1%), fear of recurrence (*n* = 280, 81%), mouth pain (*n* = 239, 69%), swallowing (*n* = 227, 65%), and speech problems (*n* = 226, 65%) most frequently endorsed (Figure 1). Decreased social contact (*n* = 73, 21%), weight loss (*n* = 96, 28%), and delayed wound healing (*n* = 106, 31%) were reported least frequently.

### Latent classes of HNC symptoms

**Figure 2.**
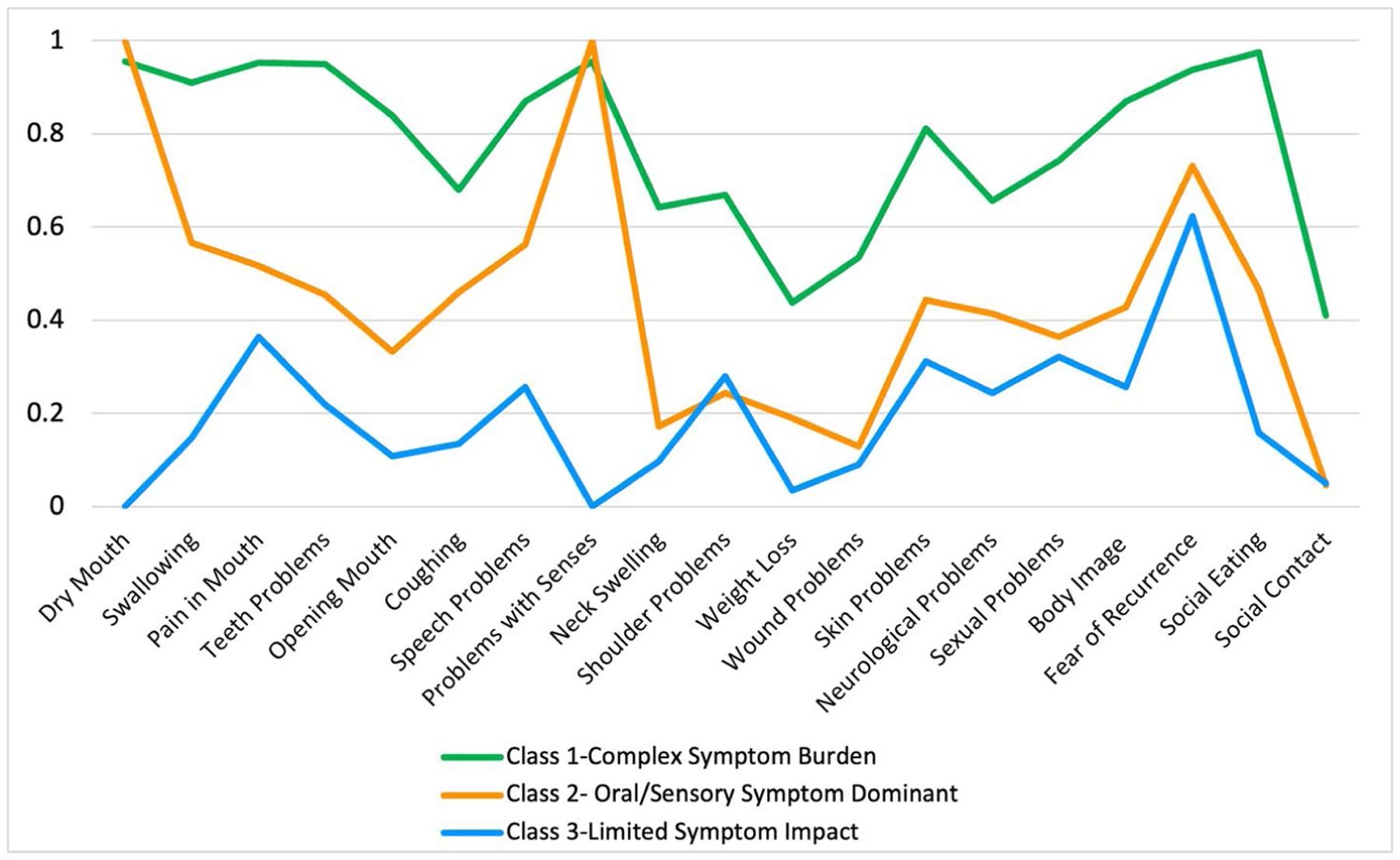
Item response probabilities for LCA of HNC symptoms. Latent class analysis (LCA) of 19 head and neck cancer (HNC) symptoms in a sample of 347 adults from Southern California, surveyed from an academic medical center tumor registry. The 19 LCA inputs were derived from the subscales of the European Organization for Research and Treatment of Cancer (EORTC HN-43) HNC module.

After comparing 2-, 3-, 4-, and 5-class models, the 3-class model was identified as the best fit based on model fit statistics (Table 2). The three latent classes were: (1) complex symptom burden, (2) oral/sensory symptom dominant, and (3) limited symptom impact (Table 3). Item response probabilities are shown in Figure 2. Forty-five percent of the sample (*n* = 155) fell into the complex symptom burden class, which had the highest number of symptoms on the EORTC-HN43 (*M* = 15, *SD* = 2). Patients in this class experienced more comorbidities (*p* = 0.01) and had a higher frequency of receipt of chemotherapy and radiotherapy (*p*<.001) and not surgery (*p*<.001) as part of their treatment history. The oral/sensory symptom dominant class included 38.9% of the sample (*n* = 135). This class was most representative of the overall sample, with an average of 8 HNC-specific symptoms (*SD* = 2). Despite fewer overall HNC-specific symptoms compared with the complex symptom burden class, this class had the highest probability among classes of reporting dry mouth and sensory problems related to smell and taste. The limited symptom impact class comprised 15.8% of the sample (*n* = 55). This group had the fewest HNC-specific symptoms (*M* = 4, *SD* = 3) and had more frequently received surgery but not chemotherapy or radiotherapy (*p*<.001) compared with the other classes. Relative to the limited symptom impact class, the oral/sensory symptom dominant class had higher frequencies of oral symptoms, such as swallowing, mouth pain, and problems with mouth opening, while psychosocial issues, such as social contact, body image, and social eating, were more comparable.

### Differences in SPADE symptoms across latent classes

**Figure 3.**
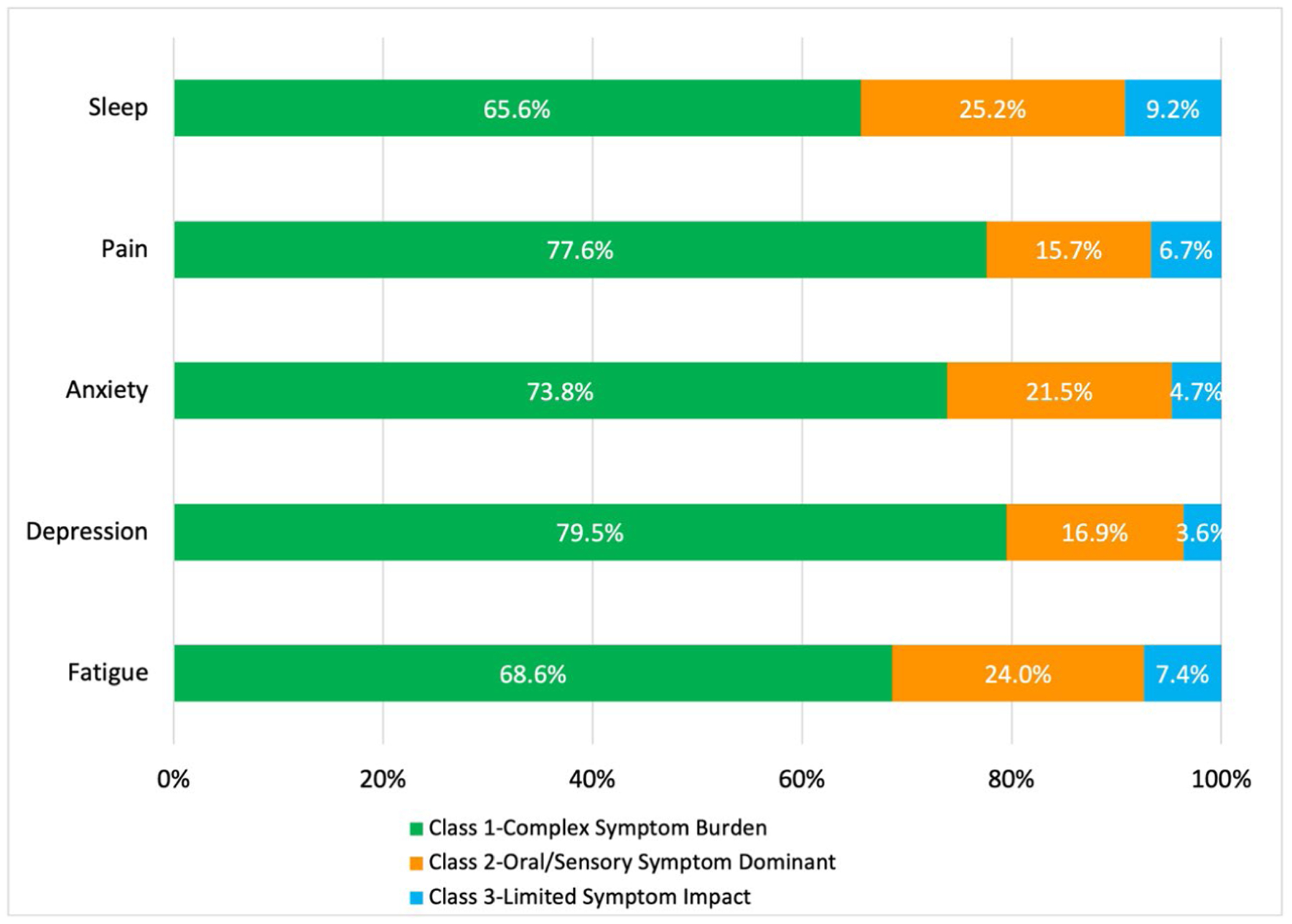
Percentage of participants with elevated levels of SPADE symptoms by latent class. Frequency of elevated levels of SPADE symptoms (sleep, pain, anxiety, depression, fatigue) among members of three latent classes, based on a latent class analysis (LCA) of 19 head and neck cancer (HNC) symptoms in a sample of 347 adults from Southern California, surveyed in 2021 from an academic medical center tumor registry. The 19 LCA inputs were derived from the European Organization for Research and Treatment of Cancer (EORTC HN-43) module on HNC, consisting of 19 subscales. SPADE symptoms were measured using Patient Reported Outcome Measurement System (PROMIS) measures and the Insomnia Severity Index (ISI).

Among those with elevated SPADE symptoms, a majority were members of the complex symptom burden class for all five SPADE categories (*p*<.001 for each SPADE category), as shown in Figure 3. Depression was most notably elevated for members of the complex symptom burden profile (80% of those with depression, *n* = 66), while members of the other two classes had relatively more frequent sleep disturbance (25% (*n* = 41) in the oral/sensory symptom dominant class and 9% (*n* = 15) in the limited symptom impact class), though not as frequent as members of the complex symptom burden class (66%, *n* = 107).

### Symptom classes and association with activities impairment and loneliness

The WPAI score indicated that regular day-to-day activities were impaired by 29.6% on average (SD: 33.6) due to health, with variation across the sample encompassing the full range from 0 to 100%. Of the overall sample, 89 (25.6%) participants were deemed lonely.

In adjusted models (Table 4), membership in the complex symptom burden class was associated with increased activity impairment (*β* = 28.6, *SE* = 3.7, *p*<.001) and increased loneliness (*β* = 1.1, *SE* = 0.2, *p*<.001) with reference to the oral/sensory symptom dominant class (which, as previously noted, was most similar to overall sample). Membership in the limited symptom impact class was not associated with activity impairment or loneliness.

## Conclusions

This study found high overlap between HNC symptom clusters, general cancer SPADE symptoms, and poor social wellbeing. Using latent class analysis, we identified distinct HNC symptom classes and found that the class characterized by complex HNC symptom burden, including high psychosocial issues (e.g. speech and communication, social participation), had the most severe quality of life impairment. Specifically, individuals in this class (38% of HNC survivors in our sample) had higher levels of SPADE symptoms and greater risk for loneliness and reduced engagement in daily activities compared with other classes. These class members more frequently had a history of chemotherapy and radiotherapy, which is consistent with other studies on loneliness in HNC patients.^22^

Our findings suggest that HNC-specific symptoms arising from cancer and cancer treatments may exacerbate other cancer-related symptoms and impair communication, self-image, and one’s ability to participate in social and community activities. Findings around loneliness are especially notable, as loneliness has been found to be a key mediator in cancer-related symptoms and social constraints and an established risk factor for mortality among cancer survivors. In addition, cancer survivors who experience loneliness appear to engage less in important health promotion behaviors in survivorship, such as physical activity, smoking cessation, and fruit and vegetable intake.^13^ There may be a bidirectional relationship between HNC symptoms and loneliness, and future studies should examine specific pathways between loneliness, HNC symptoms, and other domains of function and quality of life to inform optimal survivorship care.

Nearly half of participants fell into the complex symptom burden class. This group demonstrated the highest overall HNC-specific symptom burden, with an average of 15 HNC-specific symptoms, and the highest rates of elevated depression, anxiety, fatigue, sleep disturbance, and pain. These findings help to illuminate the complex and cumulative effects of living with multiple co-occurring symptoms on the social wellbeing of HNC survivors. Of note, this class showed the strongest associations with impairment in daily activities and loneliness, suggesting that interventions targeting symptom management may have broader social benefits. Interventions aimed at enhancing meaningful social connection and support, including structured support groups, telehealth-based counseling, social networking platforms, and activity-based programs have shown promise in reducing loneliness and improving mental health, but are currently not well integrated into cancer care delivery.^13,54^

Understanding patterns of cancer-related symptoms, treatment effects and their impact on quality of life is essential for improving survivorship care for HNC survivors. Traditional approaches to symptom analysis often overlook the co-occurrence of symptoms, instead treating each symptom as an isolated issue. However, research increasingly suggests that cancer-related symptoms tend to cluster or co-occur, interacting dynamically in ways that may intensify their impact. Prior studies of HNC symptom clusters have primarily used cluster analysis, which is a descriptive and non-model-based method, and have combined HNC-specific and general cancer symptoms in developing clusters.^55^ Our study builds upon these findings by using latent class analysis, a person-centered modeling technique that is more statistically robust than cluster analysis, to develop classes based on HNC-specific symptoms. These findings provide insights into the overlap between HNC issues, general cancer symptoms (SPADE) and dimensions of social wellbeing. Furthermore, this study indicates associations between certain patterns of symptoms and loneliness, lack of social connection, and withdrawal from daily activities.

## Implications for psychosocial providers

The identification of symptom classes with distinct social outcomes suggests the need for tailored assessment and intervention approaches based on survivors’ symptom profiles. This could include integrating routine tracking of symptoms and late complications as part of comprehensive survivorship care to identify those patients who may benefit from multimodal interventions that address both physical symptoms and psychosocial needs simultaneously. Interventions that address cancer-related symptoms and toxicities, particularly those associated with advanced disease or multimodal therapy, while fostering a sense of social connection in survivors warrant further examination in health care settings. For example, the strong association between complex symptom burden and reduced engagement in daily activities suggests the potential value of integrated occupational therapy and rehabilitation services that specifically target resumption and/or maintenance of social roles, community participation, and social connection. Future research should evaluate the effectiveness of such targeted interventions in improving both symptom management and social wellbeing outcomes among HNC survivors with different symptom profiles.

These findings have important implications for clinical practice and survivorship care delivery. The identification of symptom classes with distinct social outcomes suggests the need for tailored assessment and intervention approaches based on survivors’ symptom profiles. The strong association between complex symptom burden and poorer social functioning suggests the potential value of integrated rehabilitation services to support resumption and/or maintenance of social roles, community participation, and social connection in tandem with symptom control. Potential interventions include workplace reintegration programs that support survivors in managing treatment-related symptoms such as fatigue and cognitive impairment as they transition back to professional roles.^56–58^ Community-based wellness programs offer another promising approach, combining symptom management strategies with social engagement through group-based exercise, mindfulness or creative art therapy activities.^59–61^ Although social wellbeing itself remains largely unmonitored in clinical settings, this study underscores the link between complex symptom burden and diminished social outcomes.

This study has strengths and limitations that should be considered in understanding findings. First, it is possible that individuals experiencing higher levels of loneliness or social isolation were less likely to complete the survey, potentially limiting the generalizability of the results. As a cross-sectional self-report survey, measures may be subject to recall bias. Although the sample profile matches the demographics of HNC survivors in the US generally, future studies should examine underrepresented groups of HNC survivors to identify health disparities. The study also has notable strengths. We used validated symptom measures, including a comprehensive measure of specific HNC symptoms associated with contemporary treatments. Our study used latent class analysis, which is a methodologic improvement upon prior studies using descriptive methods.

This survey identified latent classes of HNC symptoms, with high symptom burden associated with increased general cancer symptoms (SPADE) and poorer social outcomes. Findings underscore the negative impact of severe HNC symptoms on daily functioning and social interactions, highlighting the need for tailored survivorship care models to improve overall quality of life and mitigate impacts on social wellbeing. Future research should examine pathways between HNC symptoms, loneliness, and functional outcomes to inform interventions that promote social wellbeing.

## Figures and Tables

**Table 1.** Sample demographic and clinical characteristics (*N* = 347).

Gender	*n* (%)
Female	95 (27.4%)
Male	252 (72.6%)
Race/ethnicity	
White	283 (81.6%)
Black	6 (1.7%)
Asian	21 (6.1%)
Hispanic	14 (4%)
Other	19 (5.5%)
Marital status	
Married/partnered	245 (70.6%)
Divorced/separated	54 (15.6%)
Widowed	19 (5.5%)
Single	28 (8.1%)
Household income	
≤ $60,000	73 (21%)
$60,001–$100,000	72 (20.7%)
>$100,000	161 (46.4%)
Not reported	41 (11.8%)
Insurance type	
Medicaid	139 (40.1%)
Medicare	150 (43.2%)
Commercial	58 (16.7%)
Educational attainment	
High school	142 (40.9%)
College	123 (35.4%)
Post-graduate degree	80 (23.1%)
Clinical characteristics	
Age at diagnosis	
<40 Years	16 (4.6%)
40–64 Years	191 (55%)
≥65 Years	141 (40.6%)
Charlson comorbidity Index (CCI)	
None (0)	227 (64.5%)
Mild (1–2)	104 (30%)
Moderate (3–4)	15 (4.3%)
Severe (≥5)	1 (0.3%)
Primary tumor site	
Pharynx	120 (34.6%)
Sinus, salivary, other	39 (11.2%)
Tongue, oral cavity	188 (54.2%)
Stage of disease	
Stage I	80 (23.1%)
Stage II	64 (18.4%)
Stage III	43 (12.4%)
Stage IV	103 (29.7%)
Unknown	35 (10.1%)
Time since HNC diagnosis	
2 years	71 (20.5%)
3 years	80 (23.1%)
4 years	72 (20.7%)
5 years	124 (35.7%)
Treatment history^a^	
Surgery	275 (79.3%)
Chemotherapy	164 (47.3%)
Radiotherapy	272 (78.4%)
Immunotherapy	37 (10.7%)
Targeted therapy	25 (7.2%)
Treatment group	
Surgery only	60 (17.3%)
combined chemotherapy and radiotherapy	157(45.2%)

a Treatment history categories are not mutually exclusive and reflect the exposure of a particular HNC treatment.

**Table 2.** Model fit statistics.

	AIC	BIC	LRT	*X* ^2^
2 class	7207.76	7357.883	3208.354	621,335.9
3 class	6963.105	7190.215	2923.7	706279
4 class	6979.861	7283.958	2900.456	395656.7
5 class	6827.548	7208.631	2708.143	389270.2

**Table 3.** Comparison of latent class membership.

	Overall sample (*N* = 347)	Complex symptom burden class (45.2%, *n* = 155)	Oral / sensory symptom dominant class (38.9%, *n* = 135)	Limited symptom impact class (15.8%, *n* = 55)	*P*
**Demographic Characteristics**					
Gender					0.23
Female	95	48 (50.5%)	30 (31.6%)	17 (17.9%)	
Male	252	109 (43.3%)	105 (41.7%)	38 (15.1%)	
Race/ethnicity					0.96
White	283	128 (45.2%)	109 (38.5%)	46 (16.3%)	
Non-white	60	28 (46.7%)	23 (38.3%)	9 (15%)	
Educational attainment					0.43
No college degree	142	68 (47.9%)	56 (39.4%)	18 (12.7%)	
College degree or higher	203	89 (43.8%)	78 (38.4%)	36 (17.7%)	
Marital status					0.03
Married	245	109 (44.5%)	104 (42.4%)	32 (13.1%)	
unmarried	101	47 (46.5%)	31 (30.7%)	23 (22.8%)	
Household income					0.09
<$100,000	145	72 (49.7%)	47 (32.4%)	26 (17.9%)	
>$100,000	161	66 (41%)	72 (44.7%)	23 (14.3%)	
Insurance					0.05
Medicaid	139	72 (51.8%)	49 (35.3%)	18 (12.9%)	
Medicare	150	55 (36.7%)	64 (42.7%)	31 (20.7%)	
Commercial	58	30 (51.7%)	22 (37.9%)	6 (10.3%)	
**Clinical Characteristics**					
Age at diagnosis					
<65 Years	206	84 (40.8%)	37 (18%)	85 (41.3%)	0.16
≥65 Years	141	51 (36.2%)	18 (12.8%)	72 (51.1%)	
Time since diagnosis					0.56
Year 2	71	30 (42.3%)	30 (42.3%)	11 (15.5%)	
Year 3	80	34 (42.5%)	33 (41.3%)	13 (16.3%)	
Year 4	72	30 (41.7%)	26 (36.1%)	16 (22.2%)	
Year 5	124	63 (50.8%)	46 (37.1%)	15 (12.1%)	
Primary tumor site					
Tongue and oral cavity	188	93 (49.5%)	68 (36.2%)	27 (14.4%)	0.55
Pharynx	120	49 (40.8%)	50 (41.7%)	21 (17.5%)	
Sinus, salivary, other	39	15 (38.5%)	17 (43.6%)	7 (17.9%)	
Stage of disease					0.06
I-III	191	79 (41.4%)	78 (40.8%)	34 (17.8%)	
IV	103	54 (52.4%)	40 (38.8%)	9 (8.7%)	
unknown	31	15	8	8	
Charlson Comorbidity Index					0.01
0 (None)	227	89 (39.2%)	96 (42.3%)	42 (18.5%)	
1–2 (Mild)	104	56 (53.8%)	35 (33.7%)	13 (12.5%)	
≥3 (Moderate & severe)	16	12 (75%)	4 (25%)	0 (0%)	
Treatment group					
Surgery only	59	13 (22%)	22 (37.3%)	24 (40.7%)	<.001
Combined chemotherapy and radiotherapy	157	79 (50.3%)	67 (42.7%)	11 (7%)	<.001
**Symptom Burden**					
Total HNC-specific symptoms	*M* = 10(*SD* = 4.83)	*M* = 15(*SD* = 2.16)	*M* = 8(*SD* = 2.46)	*M* = 4(*SD* = 2.8)	<0.001
Sleep	163	107 (65.6%)	41 (25.2%)	15 (9.2%)	<.001
Pain	134	104 (77.6%)	21 (15.7%)	9 (6.7%)	<.001
Anxiety	107	79 (73.8%)	23 (21.5%)	5 (4.7%)	<.001
Depression	83	66 (79.5%)	14 (16.9%)	3 (3.6%)	<.001
Fatigue	175	120 (68.6%)	42 (24%)	13 (7.4%)	<.001

**Table 4.** Association between symptom latent classes and social wellbeing outcomes.

Latent class (reference: Class 2 – oral/sensory symptom dominant)	Activity impairment score			Loneliness score		
	*β*	SE	P	*β*	SE	P
Intercept	55.3	11.2	<.001	3.9	11.1	<.001
Class 1 - Complex Symptom Burden	28.7	3.7	<.001	1.1	0.2	<.001
Class 3 - Limited Symptom Impact	0.3	5.5	0.96	−0.01	0.3	0.99

**Notes**. Multiple linear regression models estimating activity impairment and loneliness scores in a sample of 347 adult head and neck cancer survivors. Models are adjusted for age at cancer diagnosis, gender, time since diagnosis, disease stage at diagnosis, and comorbidities. SE = standard error.

## Data Availability

The data that support the findings of this study are available from the corresponding author, ERB, upon reasonable request.
